# Breaking psychedelic boundaries in public health: emerging ideas with ancient roots

**DOI:** 10.3389/fpubh.2026.1923284

**Published:** 2026-10-07

**Authors:** Kenton Bartlett, Lisa L. Gezon, Walter D. Ward, Fanicy Sears

**Affiliations:** 1 Department of Sociology, University of Alabama at Birmingham, Birmingham, AL, United States; 2 Department of Anthropology, University of Alabama at Birmingham, Birmingham, AL, United States; 3 Department of History, University of Alabama at Birmingham, Birmingham, AL, United States; 4 Department of Anthropology, University of Alabama, Tuscaloosa, AL, United States

**Keywords:** biomedical, colonization, mental healthcare, history, legalization, naturalistic use, prohibition, psychedelics

## Abstract

The modern United States faces significant public health challenges, including a mental health crisis and healthcare systems with numerous shortcomings. At the same time, emerging research on psychedelic substances demonstrates their robust applications for health and well-being, as well as their nuanced risks. While regulators explore how psychedelics can be incorporated into existing healthcare models, patterns of psychedelic use extend beyond clinical settings. These naturalistic or non-clinical applications—for spiritual growth, social bonding, ecological connection, and overall health—have occurred for thousands of years in various cultural contexts. In this critical analysis, we examine historical evidence depicting how psychedelics were utilized in the Americas, before and after European colonization. We highlight the racialized and religious origins of prohibitionist drug policy, as well as U.S. healthcare norms that constrain avenues for mental health, personal choice, and general flourishing. By drawing on interdisciplinary research in clinical mental health, history, anthropology, and medical sociology, we argue for (A.) holistic improvements to narrow mental healthcare models and (B.) an expanded-access posture towards psychedelics that includes non-clinical use scenarios. Our review of existing evidence supports these approaches for improving public mental health.

## 1 Introduction

An alarming mental health crisis in the United States (1, 2) warrants careful review of approaches to mental health and psychedelics. Exploring how humans have navigated both domains throughout history and across cultures offers unique perspectives for improvement. In this analysis, we critically evaluate clinical mental health models, public health policy, and social views around psychedelics. While preconceived notions and bureaucratic institutions present obstacles for change, historical and cross-cultural perspectives broaden one’s sense of possibility. Considering the need to balance safety and risk, we argue that the wider public stands to benefit from greater autonomy in choosing how, when, and where to responsibly use psychedelics. We also suggest that advancing holistic approaches to mental health may improve both psychedelic safety and public health. We arrive at these conclusions by conceptually reviewing interdisciplinary evidence that supports the urgent need to rethink current drug policies and healthcare models. Thoughtful engagement here is particularly relevant in countries that may be considered Westernized or part of the Global North (3). While this paper focuses on the U.S. context, public health concerns may overlap with other countries, and local policy trends around psychedelic use could have global reverberations.

To conduct this analysis, we draw from our collective backgrounds in clinical mental health, history, anthropology, and medical sociology using critical theory and medical humanities perspectives (4–6). Limitations of this review consist of both the disciplinary perspectives of the authors, combined with the scope of the literature available. We begin by foregrounding several public health challenges and the role of psychedelics within them. Specifically, we outline key limitations of biomedical and mental healthcare frameworks in the U.S., as well as the prohibition of psychedelics and their emerging medicalization. After drawing attention to related challenges, we extend our discussion across cultures and into the past. Noting variability in how psychoactive plants, fungi, and compounds are often perceived, we present evidence supporting healthy psychedelic use in several cultural settings throughout history. These include contemporary U.S. contexts, both within and beyond clinical environments (7–9), and Indigenous communities around the world (10–12). Examples of constructive use and improved health in these populations offer a counterpoint to widespread psychedelic stigma.

We conclude by underscoring the value of a medical humanities approach that critically examines basic assumptions around health, aiming to stimulate new ways of addressing persistent challenges (4, 5). Based on our assessment of the evidence, we argue that expanding access to psychedelics across a range of settings, including non-medical domains, has the potential to improve public health.

## 2 Psychedelics and health in the United States: paradigmatic tensions

This section explores how contemporary mental healthcare systems influence psychedelic care. It focuses on the limitations of predominantly biomedical approaches and considers the importance of psychological support, cultural context, community, and holistic models of healing.

Public health challenges have garnered attention for their increasing severity in recent years, alongside global health concerns (13). Among them, worsening trends in mental health have given rise to the term, “mental health crisis” (1). This phrase captures an alarming prevalence of suicide, depression, anxiety, addiction, and violence in the U.S. (2, 14–16). When reviewing these trends, healthcare experts acknowledge the limited effectiveness of prevailing interventions, including widely prescribed medications and psychotherapies (17–20). Herein lies the challenge.

### 2.1 Public health challenges and the role of psychedelics

To better understand potential drivers of these circumstances, we identify several aspects of dominant U.S. mental healthcare models that, if unaddressed, may shape the future of psychedelics in ways that affect key public health priorities, including access, equity, and community health. This overview can help evaluate critical pathways and priorities for a public health approach to psychedelics. This is particularly relevant, as psychedelics have been identified for their potential role in addressing not only mental health (7, 21) and addictive patterns of substance use (22, 23), but also physical health (24, 25), spiritual connection (26, 27), social cohesion (28, 29), and environmental stewardship (30–32).

Despite their legitimate risks and stigmatized reputation in the U.S. (33), the health and social benefits of psychoactive plants and fungi have long been recognized cross-culturally within Indigenous societies (10, 11). Contemporary scientific interest in psychedelics first emerged in the mid-twentieth century before prohibition largely halted research (34). It has since resurged following the gradual relaxation of regulatory restrictions that once again permitted clinical investigation (35). As a consequence, several psychedelics have received “breakthrough therapy” status from the FDA (36). This label is granted to drugs and medications with preliminary evidence supporting the potential to out-perform available interventions (37). While federal approval in the U.S. is pending on many psychedelic compounds, some are already available in certain states (38, 39) and around the world (40, 41). As communities respond to this evolving regulatory landscape, thoughtful engagement is needed to support public health policy.

### 2.2 Dominance of biomedicine

Confining psychedelics to healthcare settings constrains their potential to improve public health by subjecting them to medicalized restrictions (42) and healthcare’s biomedical approach (43). To clarify, “medicalization” describes how aspects of life come to be defined in medical terms, under the jurisdiction of healthcare (44). Relatedly, healthcare systems have been rooted in the biomedical model, which emphasizes body-based causes and interventions for a range of ailments (45). This model has been a cornerstone of modern medical science, accounting for significant innovations in several fields (46). Recently, however, there has been greater emphasis on approaches that recognize the importance of social, cultural, and historical contexts—particularly when addressing mental health (47, 48). While biomedical and clinical applications of psychedelic use have demonstrated many positive results in this area (7, 49), we argue for an approach that does not limit their accessibility to medical contexts. This posture aligns with policy measures in U.S. states, including Oregon and Colorado, as well as countries abroad, such as Portugal and the Netherlands (50).

Critiques of the biomedical model identify its potential to reduce the complexity of mental well-being (45, 48, 51). Additional concerns surround the model’s narrow diagnostic categories, heavy reliance on pharmaceuticals, and professional silos that separate aspects of care. Beyond this, ideologies of efficacy shape which interventions are considered safe and appropriate, or “standard of care” in clinical settings. Because psychedelics have been studied in populations considered to be “treatment resistant,” medical providers may require one to have tried antidepressant medications or traditional psychotherapy to qualify to use psychedelics, in line with clinical research (52, 53). These treatment protocols are intended to promote safety, and first-line interventions may result in benefits for many people (20, 54). They may also impose barriers to alternative forms of care (such as psychedelics) and are associated with their own risks (55–57).

Reserving psychedelics for severe mental health conditions additionally misses opportunities beyond the treatment of rigidly defined pathology (58). This includes benefits that apply to “healthy normal” individuals (8, 21, 26, 28, 30, 59–62). As emerging research highlights, motives for use in non-clinical settings are diverse (63). This varied landscape requires thoughtful consideration to support safety (64, 65) and better understand broad outcomes of well-being that are often overlooked in medical settings (66). While risks remain in both clinical and non-clinical environments, accounting for a range of legitimate use scenarios aligns with the aims of public health.

### 2.3 Clinical mental health models

Modern mental healthcare is often delivered by medical disciplines (psychiatry and nursing) and non-medical professions, including mental health counseling, psychology, and social work. We use the term “mental health models” to broadly describe the contemporary clinical mental health landscape, inclusive of both medical and non-medical fields. We also acknowledge that holistic models for mental health have been developed and implemented in some settings. For example, the biopsychosocial-spiritual model views multiple dimensions of health simultaneously (67). It highlights the interplay between body, mind, social circumstance, and spiritual or existential orientation. Because mental health and psychedelic experiences both draw on each of these domains, expansive models like these warrant consideration. Furthermore, when evaluating how psychedelics can be accessed and under what conditions, dominant approaches to mental health in the U.S. invite critical review.

#### 2.3.1 Cultural context and settings

Cultural and historical contexts influence the ways in which psychedelics are used. For example, individualized, clinical paradigms are common within biomedical healthcare systems in the U.S. (68). Engaging with psychedelics in such settings may differ from the ways psychedelics have been applied in many Indigenous cultures, as well as in non-clinical contexts, around the globe (29). These include psychedelic events that may be experienced as communal healing or spiritual ceremonies (10, 69, 70), or even as pro-social recreation (9, 71), rather than as pathology-oriented treatments or as deviant behaviors.

In contrast to neurocentric views of biomedicine that simplify psychedelics as interventions for neurological receptors, many Indigenous and non-clinical approaches consider psychedelic use to involve mind, body, nature, spirit, and community—often seen as inseparably unified (35, 72). Addressing this interconnection, psychedelic events frequently (but not always) take place outdoors, in groups, with emphasis on community support (28, 73). This can occur through emerging group models of psychedelic-assisted psychotherapy, which offer a viable path to support community-oriented psychedelic engagements in healthcare settings, with benefits around cost-effectiveness and interdisciplinary collaboration (74). Much clinical use of psychedelics, however, tends to orient around individualized interventions, facilitated in sterile indoor spaces, without communal support (29, 68). Because psychedelics are context-dependent, non-specific amplifiers, the importance of one’s immediate setting and broader social environment cannot be overstated (75).

#### 2.3.2 Models of efficacy

The relationship with a given psychoactive substance, or ideology surrounding it, also varies across culture. In a biomedical paradigm, medicine is often viewed as a remedy for sickness that is passively consumed to treat disease (48). This contrasts with many Indigenous contexts (35, 68, 69), where it is common to see psychoactive plants and fungi as teachers or entities that one respectfully listens to (31, 70). Interestingly, some psychoactive plants are referred to in anthropomorphic terms, such as Grandmother Ayahuasca or Grandfather Peyote, suggesting a consciousness or agency that resides within the organism itself (31, 76). Through this lens, a psychedelic substance does not provide healing directly. Rather, the experience offers confrontation, instruction, or support in learning to live in better alignment with health, relationships, and the larger world (35). Crucially, to receive benefits from this process, one must actively engage with these insights.

Some modalities in clinical mental healthcare share similarities, such as Inner-Directed Therapy, which is often paired with psychedelic-assisted psychotherapy (77). This approach relies on contemplation and self-reflection, rather than didactic instruction from providers. The imperative to participate in active, personal work contrasts with modern views of medical efficacy in the West, which often markets easy solutions to suffering. These notions are frequently perpetuated in the U.S. by direct-to-consumer advertising practices around pharmaceutical drugs and gratification-centered cultural norms (78, 79). Further contributing to notions of passive healthcare consumption are common views of clinicians as paternalistic decision-makers overseeing patient outcomes. These views are supported by professional trainings that encourage rigid treatment plans and meticulously defined interventions, rather than allowing space for persons to navigate healing and growth that emanate from within (69).

Building on these cultural differences, institutional forces shape mental health treatment in the modern U.S. Some of these include misaligned financial incentives that promote regularly prescribed medication, extended stays in mental health facilities, dependence on provider relationships, and billing-code conformity (80–82). Additionally, the dominant prescription model centers short, clinic-based interactions focused on diagnosis and medication, offering little support beyond the clinical timeslot. These healthcare norms present challenges for psychedelic-assisted psychotherapy, given limitations on how psychedelic substances can be administered, as well as ideologies surrounding their use.

#### 2.3.3 Balancing medical oversight and psychological safety: examples of ketamine and esketamine (Spravato^®^)

The clinical use of both ketamine and esketamine (Spravato^®^) for mental health highlights needs for integrated psychological and medical care (83, 84). Ketamine is often employed for mental health purposes within IV clinics, through at-home prescription (with and without psychotherapy), and in group, couples, and individual contexts with prioritized emotional support (85, 86). Esketamine (Spravato^®^) is an FDA-approved compound for treatment-resistant depression and suicidal ideation, commonly administered in clinical settings as a nasal spray (87). While neither substance is considered a classic psychedelic (24), they both exhibit psychoactive properties that may be considered “psychedelic” at certain doses (87, 88). As pharmaceutical companies pursue FDA approval for psychedelic substances and clinicians navigate standards of care around these compounds, safety parameters are being negotiated (87, 89). One conversation surrounds the role of psychotherapy, which is unevenly incorporated in clinical settings (85). Given that adverse events with psychedelics are more commonly psychological than medical (33, 84), we emphasize the need to prioritize non-medical aspects of care. This includes increased attention to considerations of “set and setting” (84, 87), along with the need for supportive preparation and integration (90–92).

## 3 Psychedelic history in the Americas: native practices and European prohibition

To contextualize psychedelic use patterns in the modern U.S. and imagine effective public health approaches, we draw on historical examples using the medical humanities lens. Our review focuses on the period of colonization by Europeans in the Americas. While only a fragment of psychedelic history, this under-discussed period provides context for public health discussions around psychedelic uses, mental health approaches, and regulatory postures.

During the pre-Columbian Americas (prior to 1492 CE), for example, psychoactive plants and fungi were widely used for social, political, medicinal, divinatory, and spiritual purposes (93). These included tobacco, psilocybin mushrooms, DMT snuffs, mescaline-containing cacti (peyote, wachuma), and ayahuasca (94–96). Though evidence supports their applications within Indigenous communities, the acceptance and legality of substance use in the Americas was profoundly transformed through European colonization. Considering the use of language, for instance, Europeans ascribed the term “drug” (*drogue/droga*) to a range of psychoactive and non-psychoactive plants and minerals. This encompassing word applied to naturally occurring botanicals and mushrooms with consciousness-altering effects, including ayahuasca, cannabis, datura, and psilocybin-containing mushrooms, while also describing tobacco, chocolate, cinnamon, stones, and even crushed powder from Egyptian mummies. Vocabulary of this era marked the first clear divide between licit and illicit drugs (97, 98), representing a notable contrast to Indigenous views of psychoactive plants and fungi as sacred teachers and natural medicines (11). These distinctions, often shaped by racist and colonialist assumptions, continue to influence social attitudes and legal policy around acceptable and non-acceptable substance use (97).

### 3.1 Psychedelics in the pre-Columbian Americas (prior to 1492 CE)

During this period, Spanish activity suppressed Native culture, including destroying writings which detailed traditional use of psychedelics (99, 100). We must, therefore, rely on archaeology, art, and Spanish reports to grasp how these substances were culturally integrated. Testimonies from Spanish missionaries and chroniclers must be cautiously considered, however, as they were guided by the racist and religious agendas of cultural conquest (97). Given these limitations, it is difficult to determine who used psychedelics and how access was regulated, despite ample archaeological evidence attesting to their usage. Patterns of use also varied, ranging from communal to individual, with emphasis on healing and spiritual connection.

The peyote cactus, for instance, grows along the modern U.S.-Mexico border and was consumed throughout Mesoamerica (101). Archaeological evidence indicates that peyote was used as early as 4000 BCE (102), where it was employed by priests and traditional healers for divination and healing. Closer to the Texas-Mexico border, entire communities reportedly consumed peyote together (101). A 16th century ethnographic study of Mesoamerica, *The Florentine Codex*, further notes that peyote was particularly prized amongst the chichimeca people who were “sustained” by it (103).

Elsewhere in Mesoamerica, Spanish missionaries described communal ceremonies in which priests distributed psilocybin-containing mushrooms, called *teonanacatl* or *nanacatl* in Native languages (103). These events involved fasting, dancing, drinking cacao, and singing, with the purpose of uniting communities (103, 104). Other applications of psychoactive mushrooms included use by healers and seers to divine the future and understand how to recover from illness (104, 105). Additionally, while the communal nature of mushroom-related ceremonies in this era may suggest widespread access, some accounts indicate that they were consumed only among elites—such as during the coronations of Aztec rulers (Tizoc in 1481 and Ahuitzotl in 1486) (104).

In South America, wachuma (San Pedro cacti), vilca snuff (DMT mixed with tobacco), and the plant-based brew known as ayahuasca (or yagé) were ceremonially consumed in the Andes and Amazonian jungles (101, 106). For example, at Chavín de Huántar in Peru (flourished c. 1,000–500 BCE), wachuma is widely depicted, and paraphernalia associated with vilca use was a common find by archaeologists (107, 108). Archeological sites here also include large ceremonial centers with open plazas, suggesting that rituals were communally oriented (109, 110). Without written records, access patterns remain unresolved, though psychedelics clearly played significant roles in ritual and community life (101, 111, 112).

In the South American Incan Empire, written records describe the communal nature of religious ceremonies that involved the psychoactive plant, datura (113). This plant was often infused into a corn-based alcoholic beverage, called *chicha* (114). It is believed that communities gathered within large ceremonial structures—similar to the Chavín civilization—to consume this beverage (113) Another well-documented aspect of the Incan civilization involved human sacrifice. Interestingly, the remains of human sacrifices found in southern Peru contain traces of psychedelic compounds (mescaline and harmaline), suggesting that ingesting psychedelics was also a part of these ceremonies (115). Whether individuals were given these psychedelics to reduce or increase their fears of impending death is inconclusive.

In the Amazon region, ayahuasca’s first use is unknown, with some scholars suggesting origins dating to over two thousand years ago. As recipes for ayahuasca vary by time and place, ayahuasca’s origins may be much earlier than current archaeological evidence (106, 116, 117). Relying on conservative evidence, its usage had reached the southern Peruvian coast by at least 500 CE (118). While uncertainty remains around how exactly ayahuasca was integrated in pre-Colombian times, Spanish missionaries described its applications to alleviate headaches and cure illness among Native inhabitants (119). In many tribes, only healers ingested this tea—often for medicinal and spiritual purposes—but in others it was used communally (120).

Despite the brevity of this discussion, this evidence demonstrates that psychedelics were used throughout the pre-Columbian Americas in many settings. Variations of use patterns depended on location, culture, and historical chronology. Some usages were exclusive to healers and others with specialized training. In other communities, these substances were shared with all members, thereby strengthening community connections through pro-social behaviors. Finally, in a review of access parameters, there do not seem to be prohibitions on usage in general, and when consumption was hierarchically organized around healers, priests, and rulers, details of specific restrictions remain ambiguous.

### 3.2 Licit and illicit drugs in the early modern period (c. 1500–1750)

After the arrival of Christopher Columbus to the Americas in 1492, European attitudes profoundly shaped Indigenous American usage of psychoactive substances. Importantly, Indigenous knowledge systems and traditions sharply clashed with European norms. As a result, the use of plants and fungi with psychoactive effects often evoked judgement and marginalization from colonizers. Expanding on this, several factors challenged their cultural integration. These included a rejection of Native spiritual beliefs and traditions, a lack of the kinds of community-oriented rituals that would support these practices among Europeans, and concerns about whether trade in psychedelics would be commercially profitable (120).

As Europeans evaluated these unfamiliar substances, attitudes towards psychedelics were profoundly impacted by Spanish accounts and ideology. To elaborate, Spanish observers of Indigenous practices in the Americas highlighted a sharp cultural divide, providing an unfavorable first impression to Europeans. Considering the social context, spreading Christianity was a central justification for European colonization. By extension, anything related to Native spiritual practice was viewed skeptically or deemed sacrilegious (121). As a result, Spanish laws attempted to limit consumption of psychedelics and alcoholic beverages, like *chicha*, as these were connected to Indigenous traditions. The Laws of Burgos (established in 1512 in the Caribbean and extended to Mesoamerica alongside its conquest) also prohibited the brewing of a beverage used in native religious ceremonies called *pulque*, which the Spanish believed led Native persons to commit sins and worship pagan gods (122). Further supporting this pattern, the Roman Catholic Church legally associated public intoxication with heresy and devil worship at The Second Lima Council in Peru during the 16th century (c. 1567–1568) (123).

Throughout Spanish literature in this era, negative emphasis on “drunkenness” emerges as a theme. Specifically, the Spanish word for drunk, “emborrachaa/an,” was used in early accounts to describe the effects of classic and non-classic psychedelic plants and fungi: peyote, psilocybin mushrooms (*teonanacatl*), datura (*tlapatl*), and morning glory seeds (*ololiuhqui*) (103). By equating the effects of psychedelics to “drunkenness,” these substances were condemned as devices of the devil and sacrilegious traditions of Native communities. Furthermore, the assertion that the sober and Christian-oriented culture of the Spanish was superior to other ways of life, Indigenous persons were negatively assessed as irrational, drunken, or lazy as a means of justifying social control and vilifying their ancient practices.

Specific accounts bring this into focus. For example, a Jesuit missionary, Pablo Maroni, claimed that Indigenous healers in the Amazon used ayahuasca to “consult the devil” (119). In another instance, an Indigenous man, Andrés Mixcoatl, was accused of consuming “a little mushroom, which is a demonic thing” and having “demonic” visions (105). Well-known chronicler, Fray Diego Durán (c. 1537–1588), used similar language to describe a lavish meal overseen by the Aztec ruler Motecuhzoma, where elites of Aztec society consumed mushrooms following a human sacrifice. Durán recounted that they “saw visions and had revelations about the future since the devil spoke to them in their drunken madness” (104). While scholars must critically examine Durán’s testimony for historical accuracy, his depiction of the Aztecs as a people who conducted human sacrifice and communicated with the devil through psychedelics is clear evidence of Spanish hostility towards Native spiritual practices.

In line with religious condemnation, communal events involving psychedelics were severely punished. For instance, Native persons who participated in a saint’s feast day celebration that involved psychedelic mushrooms were legally charged, resulting in mushroom use going underground until the twentieth century (124). Between 1620 and 1779, nearly one hundred cases of peyote use were additionally prosecuted by the Inquisition, further outlawing community-based ceremonies with psychedelics (101, 125).

These examples of European rejection of psychoactive substances draw attention to a sharp cultural divide that constitutes the basis for modern prohibition. Supporting this, Breen (125) argues that the cultural fabric that grounded psychedelic use in Indigenous cultures was impossible to recreate in Europe. He concludes that the intentional use of peyote, psilocybin, and ayahuasca in ceremonial contexts in the Americas “relied upon closely guarded knowledge maintained by specialized professionals. They depended upon a collective social memory and a pre-existing built environment, and they projected a grouping of ideas—about prophecy, cosmological order, and spiritual power—that were far less transferrable than the relatively universal concerns (around sociability, luxury consumption, and diet) associated with tobacco and chocolate” (125). As many of these features were associated with Native spiritual traditions, they were impossible to reconcile with European Christianity. This context remains relevant when considering the power of social forces to shape views of psychedelics and the practices surrounding them.

### 3.3 From indigenous traditions to colonial prohibition

This historical survey reveals a wide range of psychedelic use patterns and regulatory postures across time. For example, in pre-Columbian Mesoamerica and some Indigenous communities, priests and healers received specialized training that allowed them to control access to psychedelics. In these cases, psychedelics were sometimes distributed in communal ceremonies, whereas at other times they were restricted to highly trained professionals. Illegality emerged only in the early modern period, when Europe began regulating individual, communal, and ceremonial use of specific substances. These prohibitions were tied to religious and colonial projects that sought to dominate Indigenous cultures and dismantle collective psychedelic practices that supported holistic health, spiritual connection, community cohesion, and nature relatedness.

Through presenting these diverse modalities of consumption and regulation, we hope to inform a deeper understanding of the current models for medicine and mental health. Additionally, we draw attention to expanded views of constructive psychedelic use patterns—beyond restrictive clinical applications—to broaden public health possibilities.

In sum, psychedelics were widely used in the pre-Columbian Americas for healing, spirituality, and community life, with access varying from communal ceremonies to use by trained healers. European colonization introduced religious and legal prohibitions that stigmatized and suppressed many Indigenous traditions with psychoactive plants and fungi. These colonial policies helped shape modern distinctions between acceptable and prohibited substances and continue to influence contemporary attitudes towards psychedelic use.

## 4 Public health connections

Historical evidence presented here highlights several themes that apply to modern public health. First, as explained above, psychedelics were often used in community settings to support pro-social behavior, in contrast with today’s largely individual-focused clinical models. Second, they were not used to target narrow diagnoses. Rather, they were harnessed to address the whole person within social, ecological, and spiritual contexts. Third, access varied widely: some cultures restricted use to priests or healers, others incorporated guided communal practices, and some had little hierarchy at all.

This evidence of historical use patterns, alongside insights from contemporary Indigenous contexts, supports modern scholarship that suggests the value of community support. Specifically, group-based models for processing psychedelic experiences before, during, and after use have been identified for their beneficial effects (28, 74, 92). Longstanding psychedelic traditions also point to broad approaches for well-being that simultaneously address physical, social, spiritual, and mental health domains. Finally, historical artifacts underscore that access and facilitation practices vary across time and space, depending on cultural contexts. The most restrictive regulations began in the early modern period across the Americas. This era was marked by an oppressively racist, authoritarian, imperialistic, and religious agenda from European colonizers, whose influence has shaped contemporary attitudes and laws regarding psychedelics.

Acknowledging their inherent risks, these historical cases affirm research suggesting that psychedelics can be used in healthy ways in both naturalistic and clinical contexts when people have appropriate supports in place. Furthermore, historical precedent underscores the value, if not the critical importance, of transdiagnostic approaches that see beyond rigid categories of defined illness, as well as ones that consider holistic and transpersonal dimensions of human experience (58, 67, 126).

The next sections on legal landscapes, social determinants of health, and safety explore possibilities for both improving and working beyond biomedical frameworks. We advocate approaches supporting agency, autonomy, and empowerment for improving mental health outcomes.

### 4.1 Legal landscapes and medicalization

Legal framing of psychedelic access is a public health issue. Currently, these substances are controlled at national and international levels. Punctuating a long history of prohibition (emerging from European colonization across Mesoamerica), the U.S. government enacted the Controlled Substances Act in 1970 (37). This occurred in conjunction with the United Nations’ and World Health Organization’s conventions on psychotropic substance scheduling, which carried international consequences (127, 128). Relatedly, most psychedelic substances have been granted Schedule I status, indicating that they have no accepted medical use and are considered to have high potential for abuse (129). Scholars have rigorously critiqued these designations (130, 131), such as noting their anti-addictive properties which may address addictions to alcohol, cigarettes, opioids, cocaine, and other substances (22, 23). Researchers have also criticized the very notion of addiction and common views of substance use (132). Nevertheless, the restrictive classification of psychedelics relegates most use of psychedelics to discrete settings to avoid legal sanctions.

On the heels of sustained advocacy and emerging research, several pathways towards lifting prohibitions throughout the U.S. and other countries have advanced (50, 129). These efforts include ordinances, bills, ballot measures, state-based programs, as well as a presidential executive order (39, 50, 65, 133–135). These initiatives have pursued accelerated psychedelic research, FDA approval, regulation of certain types of use, and decriminalization carve-outs to decrease criminal penalties. While these moves are designed to increase availability, they often carry considerable barriers to access.

One significant limitation to legalization is the process of medicalization, which stands to relegate the use of psychedelic substances to healthcare settings as their only legal access point (136). The medicalization of psychedelics may offer benefits to many, providing a viable alternative to available interventions. This approach may also reduce stigma through demonstrating efficacy in controlled settings, rendering a more receptive culture overall and, perhaps, resulting in future legalization. However, scholars articulate important critiques of making psychedelics medically available to the exclusion of other pathways (42, 137, 138).

Concerns with medicalization are many. First, in scaling up a commodified product, regulations may undervalue the need for supporting psychotherapy. This is a trend seen with antidepressants, IV ketamine clinics, and esketamine (Spravato®) (51, 84, 139, 140). A second issue is that medicalization may actually enhance criminalization by rigidly defining contexts of proper use (in medical settings) and, by extension, also defining substance *abuse* (in non-medical environments). Among its many consequences, this diminishes potential benefits to society of non-clinical psychedelic use for relationship building, nature connectedness, spiritual growth, and personal health (8, 21). Additional motives for psychedelic engagement may surround exploring consciousness, supporting creativity, pursuing insights around oneself and the universe, investigating subconscious material, processing emotional wounds and relational ruptures, deepening appreciation of beauty, and seeking enjoyment (137). Furthermore, involving psychedelics to support spiritual and meditation practices is not uncommon (26, 141, 142). While many of these applications may improve well-being, clinical mental healthcare models narrowly view the appropriate use of prescribed medications for the treatment of mental disorders.

A strictly medicalized approach to psychedelics may place disproportionate risks and burdens on those already marginalized (32). Non-medical psychedelic practitioners, including individuals whose spiritual practices surround psychoactive plants and fungi, additionally warrant consideration. Medicalized regulations stand to cause harm to these individuals, as well as those disproportionately targeted by law enforcement. While we recognize the need for urgent healthcare innovations and supervised access options with trained professionals, the systemic and cultural issues surrounding the biomedical model must be emphasized.

In sum, legal frameworks governing psychedelic access are a significant public health issue. Unlike the programs seen in state-level psychedelic initiatives, the U.S. federal stance continues to largely restrict use to medical and research settings. While medicalization may expand access and reduce stigma, critics argue that it can reinforce inequities, criminalize non-medical use, and limit broader benefits associated with community, spiritual, and culturally grounded forms of psychedelic use.

### 4.2 Social determinants of health, access, and choice

Limiting access to psychedelics may affect social determinants of health (143). A significant body of research identifies general health risks associated with social, economic, and other disparities, as well as biases that disadvantage certain populations (144–146). Given these vulnerabilities and the diversity of individual circumstances, it is especially important that people of all backgrounds have the right to choose from a range of contexts and support avenues that address different needs. When access is gated by a strict medical model, risks and barriers to care may be unevenly distributed, with the most vulnerable groups experiencing the greatest burdens. In addition to simply increasing access to the substances themselves, there is also a need for education, both general and context-specific, about the challenges and opportunities that psychedelics provide.

In the context of psychedelics, addressing social determinants of health involves (1) cultural and trauma-attuned care that ensures socio-structural harms are not replicated in the psychedelic experience, (2) delivery models that incorporate community-based care and group-based experiences to provide equitable access, and (3) community-based and collectivist integration programming that improves affordability, connects to social services, and attunes to different cultural contexts.

One concern is the requirement for clinical support during psychedelic use. Oversight from trained professionals can be beneficial, particularly given the intensity of many psychedelic experiences and the limited availability of community-based settings in the contemporary U.S. (91). Clinical contexts may also reduce certain risks because the trust they inspire promotes engagement with established safeguards. However, clinical approaches may include protocols and assumptions that are not universally appropriate (147). In particular, legacies of colonialism, capitalism, sexism, and racism perpetuate significant harm through expressions of discriminatory power. Such bias can originate from many sources, including a healthcare provider’s personal history, cultural context, legal or professional policies, and organizational norms (144).

A second concern is that accessing psychedelics within prevailing healthcare systems may require a clinical diagnosis. This involves entrenched systems of power, where professionals trained in White-dominant education systems rely on the DSM-5—a diagnostic manual for mental disorders—to categorize and label forms of suffering (51, 143). These diagnoses often define certain presentations and symptoms as illness, while obscuring their structural causes and cultural variations. For instance, symptoms of depression may be influenced by generational trauma, systemic racism and sexism, or economic hardship caused by broad societal factors (148). However, this manual may reduce individuals to simply having “Major Depressive Disorder” and requiring isolated treatment. Not only do insurance and healthcare systems heavily rely on these labels, the implications of receiving a diagnosis are unclear. Consequences may include stigma, shifts in self-esteem, custody or legal scenario complications, employment discrimination, and other impacts on well-being (149–153).

A third issue involves the complexity of informed consent in clinical settings (154), as it may fail to account for cultural variations and associated risks (155). One significant risk surrounds uneven criminal enforcement of substance use that disproportionally targets people of color. Relatedly, drug testing may criminalize the use of substances that may have been legally acquired (156). When consent processes fail to account for the unequal risks associated with race, gender, sexual identity, and economic status, they fall short of true informed consent and the protections it is meant to provide.

A fourth matter centers around human rights and personal freedom. Specifically, the rights to freely explore one’s consciousness, pursue holistic health, and engage in spiritual activity involving psychedelics are subverted through both prohibition and restricted access parameters (157, 158). As illustrated in this analysis, humans have used psychoactive plants, fungi, and other compounds for a variety of constructive purposes for centuries (12). Historical evidence further demonstrates that these applications for spirituality and health, beyond clinical healthcare settings, are legitimate (10). The clinical environment—which is often sterile, indoors, and devoid of spiritual context—while appropriate for some, does not meet the needs of all users of psychedelics.

Thoughtful public health approaches must acknowledge this by expanding access in naturalistic settings. Evidence shows broad patterns of individual, recreational, and ceremonial use with reports of greater benefit than harm across populations (8). We argue that aligning public policy with these diverse patterns of use supports personal choice and enhances opportunities for well-being across culturally distinct populations.

Rather than advancing highly restrictive positions that unequally reproduce these harms, we call for attention to holistic and expansive models that factor in diverse cultural backgrounds and are supported by the historical examples presented above. Examples include the biopsychosocial-spiritual model (67), which accounts for one’s multidimensional social context, and legalization frameworks that empower personal agency across diverse settings (60, 159, 160). Collectively, these approaches create public health environments where cultural context is accounted for, social harms are minimized, and meaningful choice becomes possible.

In sum, restricting access to psychedelics can exacerbate health inequities by disproportionately affecting marginalized populations. Medicalized models may reinforce these inequities through diagnostic requirements, culturally narrow care, and uneven drug policy enforcement, while limiting diverse pathways to healing. We therefore advocate for holistic, culturally responsive approaches that address social determinants of health, reduce harms associated with criminalization, and expand meaningful choice.

### 4.3 Safety and moving forward

The goal of public health is to promote opportunities for well-being across populations and minimize harm. Given the limited engagement around psychedelics within public health disciplines (161), we have examined the potential of psychedelics for increasing health and quality of life, emphasizing the need for equitable access. We have also identified strengths and shortcomings of dominant models within healthcare and emerging medicalization approaches for psychedelics. Furthermore, we incorporated historical analysis to gain a broader understanding of use patterns across time and space. Analysis of psychedelic use in naturalistic, holistic, and communal settings suggests that non-clinical environments have the potential to provide safe containers for promoting individual and community well-being. The central challenge, however, is to identify the most effective way to support diverse patterns of psychedelic use in contemporary settings.

Importantly, psychedelics are not an elixir for every modern problem or appropriate for every person, and their serious risks must be accounted for (162–164). These are evocative substances, capable of producing powerful psychological, emotional, and ontological responses (26, 72, 165). While research highlights low levels of adverse events when precautions are taken (33), the nature of their effects remains a concern—particularly in vulnerable populations.

To support optimal outcomes, safety must be considered in all psychedelic contexts, including research (166), clinical (167), and naturalistic settings (8, 89). This includes diverse opportunities for emotional support and safe access to these substances, without risk of chemical adulterants or legal sanctions. Beyond the act of taking a substance, particular attention must be paid to preparation for and integration of these potent experiences (90). We emphasize this as social and psychological support have been acknowledged for their crucial role in reducing harm and optimizing benefits (91, 92).

Even if these safety measures were perfectly implemented, however, the likelihood of adverse events can be exacerbated by negative public opinion and regulatory policy that obscure their potential benefits. Given the vital role of social context (75), addressing myths, misinformation, and residues of prohibition must be prioritized to support holistic psychedelic safety. Confronting these challenges requires ongoing education, as well as thoughtful engagement surrounding psychedelic substances and their associated policies (138). Through these pathways, we suggest that adverse events can be minimized and public health can flourish.

## 5 Conclusion

Urgent challenges around mental health and healthcare systems invite modifications of dominant approaches to mental well-being. This critical analysis explores these issues through interdisciplinary perspectives, focusing on access to psychedelic substances.

While the use of psychedelics in clinical settings has significant potential to benefit many, exclusively relegating these substances to medicalized environments has numerous drawbacks. Acknowledging that stigma often surrounds psychedelic use in cultures that have not widely integrated them, historical and contemporary evidence supports that psychedelics can be safely utilized across many contexts. Intentions for use often surround mental-health support, spiritual growth, and emotional processing, as well as deepening relationships, connecting with nature, and exploring consciousness through insight-oriented practices. Though serious risks must be acknowledged, research highlights beneficial outcomes across populations.

In sum, our modern critique and historical review point to rethinking policies and regulations that overestimate the benefits of modern mental health approaches and outlaw the safe use of psychedelics. This review instead supports holistic models and expanded-access policies for safe and legal psychedelic use to secure the public’s health.
